# Ambient nitrate switches the ammonium consumption pathway in the euphotic ocean

**DOI:** 10.1038/s41467-018-03363-0

**Published:** 2018-03-02

**Authors:** Xianhui Sean Wan, Hua-Xia Sheng, Minhan Dai, Yao Zhang, Dalin Shi, Thomas W. Trull, Yifan Zhu, Michael W. Lomas, Shuh-Ji Kao

**Affiliations:** 1 State Key Laboratory of Marine Environmental Sciences, Xiamen University, 361101 Xiamen, China; 2 Antarctic Climate and Ecosystems Cooperative Research Centre, University of Tasmania, and CSIRO Oceans and Atmosphere, Hobart, 7001 Australia; 3 Bigelow Laboratory for Ocean Sciences, East Boothbay, ME 04544 USA

## Abstract

Phytoplankton assimilation and microbial oxidation of ammonium are two critical conversion pathways in the marine nitrogen cycle. The underlying regulatory mechanisms of these two competing processes remain unclear. Here we show that ambient nitrate acts as a key variable to bifurcate ammonium flow through assimilation or oxidation, and the depth of the nitracline represents a robust spatial boundary between ammonium assimilators and oxidizers in the stratified ocean. Profiles of ammonium utilization show that phytoplankton assemblages in nitrate-depleted regimes have higher ammonium affinity than nitrifiers. In nitrate replete conditions, by contrast, phytoplankton reduce their ammonium reliance and thus enhance the success of nitrifiers. This finding helps to explain existing discrepancies in the understanding of light inhibition of surface nitrification in the global ocean, and provides further insights into the spatial linkages between oceanic nitrification and new production.

## Abstract (web summary)

The underlying regulatory mechanisms of phytoplankton assimilation and microbial oxidation of ammonium in the surface ocean are unclear. Here, using isotope labeling experiments, the authors show that ambient nitrate is a key variable bifurcating ammonium flow through assimilation or oxidation.

## Introduction

Ammonium (NH_4_^+^) is a central component in the marine nitrogen cycle. The relative flow of NH_4_^+^ through assimilation by phytoplankton versus oxidation by microbial nitrifiers largely determines the composition of the upper ocean nitrogen pool, which plays a crucial role in controlling marine productivity and the export of fixed carbon in the ocean^1^. The pathway to organic nitrogen through assimilation represents regenerated production, while the pathway to nitrate (NO_3_^−^) through nitrification fuels new production and produces the greenhouse gas nitrous oxide (N_2_O). The two competing pathways are known to show spatial separation in some oceans, where nitrification occurs deeper than ammonium assimilation, and this separation has long been assumed to be light-modulated^2, 3^. However, this view is challenged by recent evidence of active nitrification in the sunlit ocean^4–8^. Intriguingly, surveys on distributions of nitrification in the global sunlit ocean exhibit paradoxical results. In the subtropical North Atlantic, nitrification in the euphotic zone appears to be inactive during winter when light irradiance is low^9, 10^ and becomes more active in late spring and summertime with higher light intensity^4, 5^, resulting in a counterintuitive light effect on nitrification. In the North Pacific, nitrification remains undetectable in the entire euphotic zone of the subtropical gyre, but is prominent even at the surface in high latitude and equatorial areas where subsurface waters outcrop^6^. These inconsistencies indicate additional controls on nitrification are likely.

Nutrient concentrations are another fundamental environmental parameter influencing biogeochemical processes. They exert primary control on species composition, cell size structure, and associated nutrient uptake capacity of phytoplankton assemblages in the ocean^11–15^, with significant variation in uptake and growth kinetics of phytoplankton communities occurring under different nutrient conditions, including increasing half-saturation concentrations from oligotrophic to nutrient replete regions^16^. Thus, we hypothesize the nutrient status to be an essential controlling factor on NH_4_^+^ flows in upper ocean waters through regulating the relative competitiveness of phytoplankton and nitrifiers under limiting levels of NH_4_^+^.

To explore controls of NH_4_^+^ cycling by the interactive relationships between phytoplankton and nitrifiers, simultaneous rate measurement of NH_4_^+^ assimilation and oxidation is necessary; surprisingly, such data remain sparse. To our knowledge, direct comparison of NH_4_^+^ utilization capabilities between phytoplankton and nitrifiers is missing to date in both laboratory and field studies.

To address this gap and to unravel the biogeochemical implications of ammonium pathways in dynamic oceans, we measured simultaneously NH_4_^+^ uptake, NO_3_^−^ uptake, and nitrification rates, at sites spanning a wide range of nutrient conditions from coastal to open ocean waters in the South China Sea and the Northwestern Pacific Ocean (Supplementary Fig. 1). By using isotope labeling techniques and a refined rate calculation approach (see Methods), we obtained multiple rate profiles with high vertical sampling resolution, which allowed us to capture the sharp gradients in biological processes and associated physical-chemical conditions in the upper ocean.

Moreover, for the first time, we compared the competitive capabilities of nitrifiers and phytoplankton by testing their underlying ammonium utilization kinetics (specifically, the potential maximum reaction rate *V*_max_, the half saturation concentration *K*_s_, and the substrate affinity *α*)^12, 17^, at varying light and nutrient levels. This kinetic information provides robust evidence regarding the competitive advantages of nitrifiers versus phytoplankton for NH_4_^+^, under different environmental conditions. We find that ambient NO_3_^−^ level, which regulates phytoplankton’s reliance and affinity toward NH_4_^+^, is a key variable to bifurcate NH_4_^+^ flow through assimilation versus oxidation.

## Results

### NH_4_^+^ uptake and nitrification rates

**Fig. 1:**
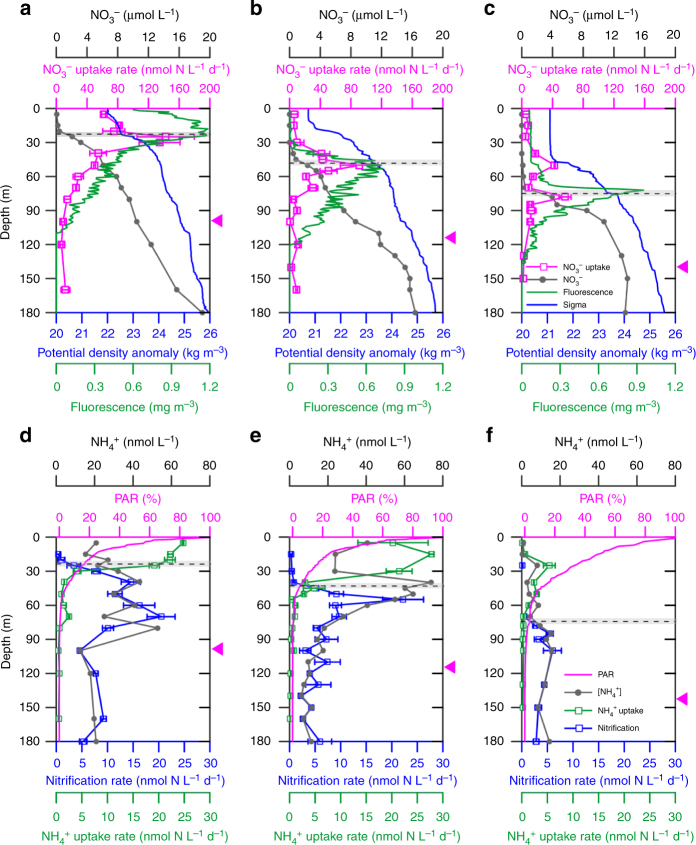
Depth profiles of physical-chemical parameters and NH_4_^+^ consumption rates in the South China Sea in the summertime of 2014. **a**, **d** Station D1; **b**, **e** Station H3; **c**, **f** Station X2. Panels **a**−**c** show NO_3_^−^ concentrations (gray solid dots); NO_3_^−^ uptake rates (pink open squares); potential density anomalies (blue line) and chlorophyll fluorescence values (green line). Panels **d**−**f** show NH_4_^+^ concentrations (gray solid dots); percent of surface PAR (pink curve); NH_4_^+^ uptake rates (green open squares), and nitrification rates (blue open squares). Gray bars mark the depth range with the steepest NO_3_^−^ gradient and the black dashed lines within them show the nitracline depth; pink triangles on the right *Y*-axis mark the euphotic zone depth (0.1% of surface PAR). Error bars of NH_4_^+^ uptake rates, NH_4_^+^ oxidation rates, and NO_3_^−^ uptake rates are represented by the standard deviation of duplicates, and are smaller than the symbols where not visible

In the South China Sea, all three stations (X2, H3, and D1) displayed typical shallow stratification of the subtropical oligotrophic ocean in warm seasons. Based on a temperature threshold criterion with a difference of 0.8 °C from the surface value^18^, we found that X2 held the deepest and strongest stratification with mixed layer depth of 50 m. Station H3 showed the second strongest stratification despite it held mixed layer depth of around 20 m similar to D1 (Supplementary Fig. 2). By defining the mid-point (average) of the depth range with the steepest gradient as the location (depth) of the nitracline^19^, we found the depths of the surface mixed layer and the nitracline co-varied. As the nitracline shoaled, the deep chlorophyll maximum (DCM) migrated upward accompanied by higher Chl-a (Fig. 1a–c) and higher particulate organic nitrogen (PON) (Supplementary Fig. 2). Corresponding to such migration, the light penetration depth was influenced by the biomass (see PON levels in Supplementary Fig. 2) with attenuation coefficients of 0.07 at D1, 0.06 at H3, and to 0.05 for X2. Here, the euphotic zone was defined by the 0.1% surface photosynthetically active radiation (sPAR) depth, because low levels of NH_4_^+^ uptake were observed below the 1% sPAR level. The euphotic depth thus deepened from 95 m at D1 to 115 m at Station H3 and 140 m at X2, being positively correlated with the nitracline depth. NH_4_^+^ concentrations were low (1–74 nmol L^−1^) without clear vertical maxima. By contrast, primary nitrite maxima (PNM, with maximal concentrations ranging from 185.5 to 254.0 nmol L^−1^) were observed slightly above the 1% sPAR depth and below the DCM for all stations. NO_3_^−^ was depleted in the mixed layer for all stations (<0.2 μmol L^−1^) and remained low downward until the depth of the gradient-defined nitracline. Interestingly, below the DCM layer at all stations there were distinct NO_3_^−^/SiO_4_^−4^ maxima with peak values ranging from 1.1 to 1.2 (Supplementary Fig. 2), suggesting preferential remineralization and nitrification of N in sinking particle had occurred^20, 21^.

The paired light and dark incubations provide direct evidence of light effects on phytoplankton and nitrifiers. Nitrification rates were significantly higher in the dark than in the light at all sites (*p* < 0.05, Supplementary Table 1); the inhibition effect was most prominent in the upper euphotic zone and decreased downward with the attenuation of light (Supplementary Fig. 3a–c). Nevertheless, detectable rates (higher than the detection limit of 0.04–0.16 nmol N L^−1^ d^−1^, see Methods) remained in the dark incubations in the upper euphotic layer at most of our study sites, indicating recovery of ammonia oxidizers from photo damage. In other words, the efficiency of nitrification was substantially reduced but not completely inhibited within a diel cycle. Conversely, light enhanced NH_4_^+^ uptake was low in the dark (Supplementary Fig. 3d–f), although the differences between light and dark rates were not as significant as for nitrification (Supplementary Table 1). Thus our results also reinforce the necessity of paired light and dark incubations to obtain precise reaction rates.

The most striking feature of the NH_4_^+^ uptake and nitrification rate profiles was their inverse unimodal distributions, with assimilation above and nitrification below a remarkably strong transition boundary at the nitracline, regardless of its depth or irradiance level (Fig. 1d–f, Supplementary Fig. 4). This implies ambient nitrate might play an important role in niche partitioning of NH_4_^+^ assimilation versus oxidation. NH_4_^+^ uptake was active above the nitracline with peak values ranging from 5.4 to 27.8 nmol N L^−1^ d^−1^ for the three stations. Dramatic downward decreases (reduction by 73−82% within 20 m) in NH_4_^+^ uptake were found near the nitracline, and the rate remained detectable throughout the lower euphotic zone. By contrast, nitrification was low or undetectable in the mixed layer and increased rapidly downward around the nitracline, peaking (with maximum rates of 5.9−22.2 nmol N L^−1^ d^−1^) near the PNM and upper oxycline. Below the depth of the nitrification maximum, rates decreased gradually toward greater depth. The vertical distribution pattern of nitrification broadly agrees with current recognition that nitrification is fueled by remineralization of organic matter^22^ and contributes a considerable amount of NO_2_^−^ to form and maintain the PNM^23, 24^. The values of maximum NH_4_^+^ uptake and nitrification were comparable, suggesting both pathways actively contributed to the consumption of NH_4_^+^, but in separate domains in the sunlit oligotrophic ocean.

**Fig. 2:**
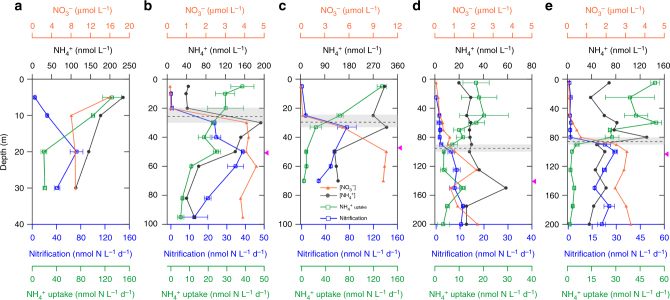
Depth profiles of nutrient concentrations and NH_4_^+^ consumption rates in the Subtropical Northwest Pacific in spring of 2015. **a** coastal station P1; **b**, **c** Shelf stations P5 and C5; **d**, **e** basin stations K1 and X1. The figure shows NO_3_^−^ concentrations (orange solid dots); NH_4_^+^ concentrations (gray solid dots); NH_4_^+^ uptake rates (green open squares) and nitrification rates (blue open squares). Gray bars mark the depth range with the steepest NO_3_^−^ gradient and the black dashed lines within them show the nitracline depth; pink triangles on the right *Y*-axis mark the euphotic zone depth (0.1% of surface PAR). Error bars of NH_4_^+^ uptake and oxidation rates are represented by the standard deviation of triplicates, and are smaller than the symbols where not visible

To further examine the applicability of the observed relationship between nitrate concentration and NH_4_^+^ consumption pathways, we measured NH_4_^+^ uptake and nitrification rates in the Northwest Pacific Ocean. Five stations across coastal, shelf, and open ocean conditions, with large gradients in surface nutrients and biological activities were selected (Supplementary Fig. 5). The coastal station showed significantly higher nutrient and biological activities (inferred from fluorescence and PON) while the open ocean stations all showed shallower nitraclines and lower biomass (Supplementary Fig. 5). For the shelf (P5 and C5) and basin (K1 and X1) stations with shallow stratification, the transition boundaries from NH_4_^+^ uptake dominance to oxidation dominance were observed in every case to be near the corresponding nitraclines (i.e. at the same depth within the site specific 5–10 m resolution of the sampling). At the eutrophic coastal station P1, where no nitracline was present, NH_4_^+^ uptake and nitrification co-occurred throughout the water column (Fig. 2), similar to results observed in Monterey Bay^7^. These results from a wide range of environmental conditions demonstrate a ubiquitous regulatory influence of nitrate on the competition for NH_4_^+^ between phytoplankton and nitrifiers.

### NH_4_^+^ utilization capabilities

**Fig. 3:**
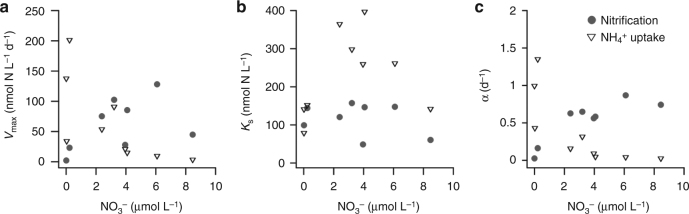
The kinetic traits of phytoplankton and nitrifiers under different NO_3_^−^ conditions in the SCS. **a** The potential maximum reaction rate (*V*_max_); **b** the half saturation concentration (*K*_s_); **c** the substrate affinity (*α*). Solid circles represent kinetic traits of nitrification and open triangles represent NH_4_^+^ uptake

We investigated the dependence of nitrification and NH_4_^+^ uptake rates on substrate concentration by adding different amounts of isotopic-tracer-labeled substrate, providing a method that calibrates enhancement of rates by tracer enrichment^25^. The observed kinetic responses also enable us to compare the competitive capability of these organisms towards the limiting nutrient^12, 17^. Values of *V*_max_ for nitrification were significantly higher than NH_4_^+^ uptake below the nitracline where NO_3_^−^ was replete (*p* < 0.05) (Fig. 3a, Supplementary Fig. 6 and Supplementary Table 2). The *K*_s_ for nitrification did not show a clear vertical trend, whereas the *K*_s_ for NH_4_^+^ uptake by phytoplankton exhibited values below the nitracline higher than above, although with strong variability (Fig. 3b). Accordingly, a clear difference between nitrifiers and phytoplankton in affinity (*α*) toward NH_4_^+^ was observed under different NO_3_^−^ concentrations, the *α* of phytoplankton was higher above the nitracline while the *α* of nitrifiers was higher below (*p* < 0.01) (Fig. 3c, Supplementary Table 2). These differences in substrate affinity above and below the nitracline obviously reflect the relative competitive advantages between phytoplankton and nitrifiers.

## Discussion

Our results suggest light inhibition of nitrifiers is not the single determinant for the distribution of nitrification in the surface ocean. In spite of the inhibition of ammonia oxidizers by light via photo damage to the key enzyme ammonia monooxygenase, as well as other membrane components^26^ or the toxicity of reactive oxygen species^27^, some taxa of marine AOA have been identified as having genetic capabilities to reduce oxidative stress and repair ultraviolet damage^28, 29^ and thus appear to be resistant to light in the upper ocean^30, 31^. Consistent with these perspectives, our paired incubations showed that light-inhibited nitrifiers either recovered in the dark or some portion of their population was light resistant (Supplementary Fig. 3a–c). Consequently, detectable nitrification rates were observed even in the surface layer (Supplementary Fig. 4). This is consistent with reported nitrification rates in the euphotic zones of a wider range of environments, which all exhibit resistance to light inhibition^6–8, 24, 25^.

The co-variation of light and NO_3_^−^ in the stratified oligotrophic ocean often obscures differentiating their effects on regulating euphotic nitrification. Nevertheless, our measurements with high vertical resolution combined with the kinetic tests allowed us to distinguish the role of NO_3_^−^ from light and thus to give a more comprehensive evaluation of the underlying mechanisms of NH_4_^+^ consumption. Unexpectedly, we found that the nitrification rates have stronger correlations with NO_3_^−^ concentrations than with light levels, from the surface to the depth of the rate maxima (Supplementary Fig. 7). Moreover, we observed the depth transition from NH_4_^+^ uptake to nitrification was highly consistent with the depth of the nitracline (Figs. 1, 2, Supplementary Fig. 4), even though light intensity at this depth varied among the stations. These results suggest a previously unrealized ecological control of NO_3_^−^ in structuring the spatial domains of nitrifiers.

According to our kinetic tests, the nitrifier and phytoplankton communities had similar *K*_s_ values for NH_4_^+^ in layers above the nitracline (Fig. 3b), and these values were consistent with field and laboratory studies^25, 32, 33^. However, the *V*_max_ and *α* values were higher for phytoplankton above the nitracline (Fig. 3a), resulting in a competitive advantage of phytoplankton toward NH_4_^+^. Light stimulates phytoplankton growth, and dark does not damage these cells, and accordingly the phytoplankton showed significantly higher *V*_max_ than nitrifiers over the diel cycle. Phytoplankton thus outcompete ammonia oxidizers to be the main NH_4_^+^ consumer in the surface mixed layer. Below the nitracline, by contrast, significantly higher values of *V*_max_ and *α* for nitrification evidence a competitive advantage for nitrifiers over phytoplankton (*p* < 0.05, Supplementary Table 2). This was due to the benefit of reduced light inhibition on nitrifiers (Supplementary Fig. 3) as well as the reduction in affinity of phytoplankton toward NH_4_^+^.

In fact, our rate profiles demonstrate a downward shift of nitrogen source for phytoplankton from NH_4_^+^ to NO_3_^−^. The NO_3_^−^ uptake rates showed an increasing trend with depth and peaked around the nitracline, and the rates remained significantly higher than NH_4_^+^ uptake rates below the nitracline at all sites (*p* < 0.05). Similar results of higher NO_3_^−^ than NH_4_^+^ uptake rates have also been observed at BATs during the deep mixed layer season^5^. Because NO_3_^−^ utilization is more energetically costly, the low NH_4_^+^ uptake rates were unlikely to be caused by light deficiency, an alternative possible cause is the high nutrient and low light conditions, which often lead to eukaryotic phytoplankton dominance. Previous studies have shown the interactions of NH_4_^+^ and NO_3_^−^ uptake by phytoplankton appear to be highly diverse and vary with changes in species, preconditioning (nutrient history) and light^34–36^. For example, incubation of diatoms under low light shows high NO_3_^−^ uptake rates in spite of high NH_4_^+^ enrichment^37^. Our results are also supported by short-term experiments that find phytoplankton are less reliant on NH_4_^+^ as their nitrogen source, when pre-conditioned with enriched NO_3_^−^ (compared to preconditioning with NH_4_^+^)^34^, and with no inhibition of NO_3_^−^ uptake by NH_4_^+^ under low light, possibly due to acclimation to satisfy nitrogen requirements for cell growth^35^ or because of enzymatic rather than energetic limitation of growth^36^. Analogous subsurface NO_3_^−^ uptake maxima have also been observed in the North Atlantic subtropical gyre, associated with elevated abundances of picoeukaryotes, implying their active uptake of NO_3_^−^^38^, which is also evidenced by the δ^15^N of sorted picoeukaryotes^39^. Moreover, our NO_3_^−^ uptake rates showed significant positive correlation with fluorescence at all our study sites (*p* < 0.01, *R*^2^ = 0.63, Supplementary Table 3), providing additional evidence that the phytoplankton communities below the nitracline were predominantly dependent on NO_3_^−^ (Fig. 1a–c). Comprehensive understanding of the distinctive and variable kinetic responses of NH_4_^+^ and NO_3_^−^ uptake to varying light and nutrient conditions and histories remains unclear, but the spatially decoupled maximum *V*_max_ of NH_4_^+^ uptake (at 15 m) and NO_3_^−^ uptake (around the nitracline) demonstrate strongly divergent nutrient utilization strategies for the phytoplankton communities living in different nutrient conditions. Collectively, these results suggest that the downward increase of ambient NO_3_^−^ concentration has an important role in modulating the relative competitiveness of phytoplankton and nitrifiers for NH_4_^+^, likely via its influence on phytoplankton community structure.

This mechanism for reduced phytoplankton reliance on ammonium at depth as a result of structuring of the community by nutrient availability is consistent with broader ecological theory in which ambient nutrient conditions are regarded as the most important factor in determining the structure and mean cell size of phytoplankton communities in marine environments^14, 15, 40^, with oligotrophic regions dominated by phytoplankton with small cell size, and larger cells present in nutrient-replete regions. Consistent with this generality, transition from prokaryote dominance of the phytoplankton community in the mixed layer to eukaryote dominance below the nitracline was evidenced by pigment data in the SCS (for instance, in the H3 station of our cruise, prokaryotic phytoplankton account for 85.5 ± 0.1% of total biomass in the mixed layer and the ratio of eukaryotes increase to 67.8 ± 2.9% from DCM to the bottom of euphotic zone, Supplementary Fig. 8). Dominance of *Prochlorococcus* in the mixed layer and increase of eukaryotes near the nitracline has been previously observed at other locations in our study area^41, 42^ as well as other low latitude oligotrophic oceans^43, 44^. *Prochlorococcus* preference on NH_4_^+^ is consistent with short-term incubations of flow-cytometrically sorted *Prochlorococcus* populations, of which NO_3_^−^ was found to account for only <10% of their nitrogen source^45^, despite their genetic capability to utilize NO_3_^−^^46^. By contrast, eukaryotic phytoplankton often show higher nutrient demand and higher nutrient *K*_s_ values due to their larger cell size^11, 13, 47, 48^. This is consistent with our results showing that the *K*_s_ of NH_4_^+^ uptake increased from the NO_3_^−^-depleted surface waters downwards into the NO_3_^−^-replete depths (Fig. 3b), and indicates lower affinity on NH_4_^+^ in the phytoplankton in the high NO_3_^−^ environment. This perspective of ambient nutrient control of community structure is further supported by studies showing that increases of NO_3_^−^ substantially stimulate biomass production and push phytoplankton communities towards large diatom dominance^49–51^. Consequently, nitrification is likely to be enhanced due to alleviation of competition for NH_4_^+^ from phytoplankton. However, to our knowledge, direct evidence of stimulation of nitrification by NO_3_^−^ enrichment, via shifts in phytoplankton community structure, remains unexplored in the field and thus warrants more verification in future.

**Fig. 4:**
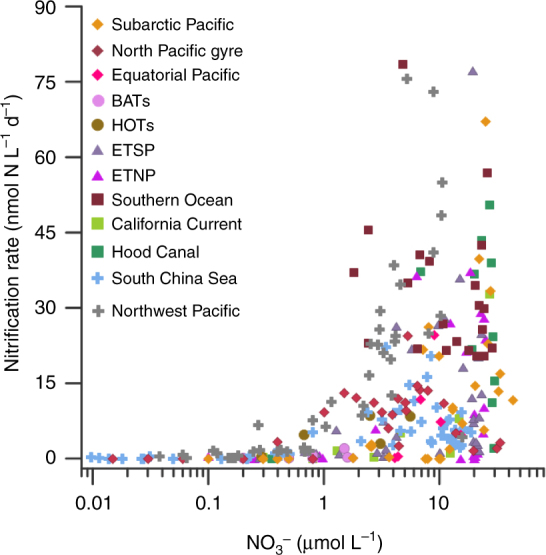
Nitrification rates versus NO_3_^−^ concentrations in the upper 200 m of different ocean provinces. Data of Subarctic Pacific, North Pacific gyre, and Equatorial Pacific are from ref.^6^; BATs from ref. ^10^; HOTs from ref. ^4^; ETSP from ref. ^67^; ETNP from ref. ^66^; Southern Ocean from ref.^69^; California Current from ref. ^24^; Hood Canal from ref. ^25^; South China Sea and Northwest Pacific from this study

Our results also help to elucidate the causes of the discrepancies in the extent of surface nitrification in different oceanic regimes. By compiling reported nitrification rates in the upper 200 m of different ocean provinces, we found persistently low rates of nitrification above the nitracline, and that high rates were always associated with NO_3_^−^-replete conditions, in spite of the highly varying locations of the observations (Fig. 4). In the cases with deep nitraclines, nitrification rates were low throughout the euphotic zone, likely due to intense competition for NH_4_^+^ by phytoplankton under NO_3_^−^-depleted conditions (Supplementary Note 1). Conversely, in nutrient-replete coastal or upwelling regions, active nitrification has been reported even in surface layers with high light intensity^7, 8, 52, 53^, demonstrating a niche in high-nutrient environments for nitrifiers. This close relationship between nitrification and NO_3_^−^ suggests an obligatory condition of NO_3_^−^ for nitrification to occur in the sunlit ocean, which reconciles the discrepancies in the apparent light effects on nitrification for different ocean provinces.

**Fig. 5:**
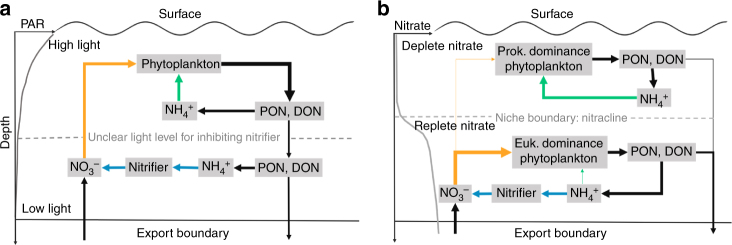
Conceptual diagrams for the regulation of ammonium transformation pathways. **a** Conventional view: the control of the vertical distribution of NH_4_^+^ assimilation and nitrification in the euphotic ocean is separation by light: phytoplankton dominate NH_4_^+^ consumption at high-light intensity, and reduced light inhibition enables nitrifiers to dominate NH_4_^+^ consumption at greater depth. However, the light level for inhibition shows large temporal-spatial variations and thus the niche boundary for nitrifiers is unclear. **b** Amended view: ambient NO_3_^−^ exerts important influence on the competition for NH_4_^+^ by nitrifiers versus phytoplankton via determining phytoplankton community structure, and thus, the affinity of phytoplankton for NH_4_^+^. Above the nitracline, prokaryotes dominate, yielding higher affinity for NH_4_^+^; while below the nitracline eukaryotes dominate and use the abundant NO_3_^−^ as the main nitrogen source, reducing phytoplankton affinity for NH_4_^+^ and enabling nitrifiers to dominate ammonium recycling. Thus, the depth of the nitracline represents a predictable boundary for the bifurcation of the fate of ammonium. Blue arrows indicate nitrification; green and orange arrows indicate NH_4_^+^ uptake (regenerated production) and NO_3_^−^ uptake (new production), respectively

Conventionally, nitrifiers and phytoplankton are thought to be separated by light, phytoplankton dominates NH_4_^+^ consumption in high irradiance environments, and nitrification is driven by mineralization of PON and DON in low-light conditions, yet, whether there is a threshold light value for setting the niche boundary of nitrifiers has been unclear (Fig. 5a). Our evidence underscores that light is not the sole determinant and that the ambient NO_3_^−^ concentration exerts an important influence. Based on our results, the depth of the transition boundary of NH_4_^+^ uptake and nitrification was consistent with the nitracline at all stratified oligotrophic sites, suggesting the depth of the nitracline represents a reliable boundary separating the space domains of ammonium oxidizers from assimilators (Fig. 5b). This finding extends our current knowledge of regulatory mechanisms for nitrification in the stratified oligotrophic ocean.

Finally, euphotic nitrification has important implications regarding bottom-up NO_3_^−^ supply versus oceanic new production^54^. The observed inter-relationships among NO_3_^−^ concentration, phytoplankton community structure, and the optimal niche for nitrifers, bind nitrification tightly with new production inside the euphotic zone. Specifically, the NH_4_^+^ assimilators dominated in the mixed layer, while NO_3_^−^ assimilators and nitrifiers developed in response to the downward increase of NO_3_^−^ (Supplementary Fig. 9a). Thereby, a predictable relationship between nitrification and new production is formed, the ratio of nitrification rate to new production rate increases from the surface to the bottom of the euphotic zone (Supplementary Fig. 9b). Notably, in our study the *V*_max_ values of nitrification at the DCM and 1% PAR layers reached the same magnitude as NO_3_^−^ uptake rates (Supplementary Fig. 7). This suggests that equating nitrate uptake with new production is an over-simplification, and also that nitrifiers were substrate stressed and thus potentially capable of even greater dominance of ammonium recycling and nitrate supply.

The ongoing warming of the global ocean is expected to enhance ocean stratification, and thus may have significant impacts on the cycling of NH_4_^+^ by influencing the supply of subsurface NO_3_^−^. For instance, global decline of phytoplankton and production accompanied by increases of niches for cyanobacteria *Prochlorococcus* and *Synechococcus* due to the climate-driven expansion of the stratified oligotrophic ocean^55, 56^ would likely depress nitrification through less supply of NO_3_^−^ to the euphotic zone and more intense competition of NH_4_^+^ by the small size phytoplankton. This migration of nitraclines would also influence the spatial domains available to nitrifiers. Thus the future changes in ocean stratification may cause reallocation of spatial domains for both active NH_4_^+^ assimilators and oxidizers. In view of the important role of NH_4_^+^ in marine nitrogen and carbon cycling, our study helps to understand the nitrogen pathways in the sunlit ocean and improve our ability to predict how global change is likely to impact the nitrogen cycle and in consequence the ability of the ocean to absorb CO_2_.

## Methods

### On-deck incubations

Samples were collected from June to July 2014 in the South China Sea (SCS) and from April to May 2015 in the Subtropical Northwest Pacific (NWP) by R/V *Dongfanghong II*. Three stations spanning a range of hydrographic conditions and corresponding biological productivities from shelf to the basin in the SCS, and five stations spanning from coastal waters to the subtropical gyre of the NWP with even larger environmental gradients were chosen for on-deck incubations (Supplementary Fig. 1).

Temperature, salinity, and chlorophyll concentrations were measured using a Seabird SBE 911 CTD sensor package equipped with a fluorometer. Photosynthetically active radiation was measured using a PAR sensor (Li-Cor Biosciences, LI-193). Samples were collected using 12-L Niskin bottles mounted on the CTD rosette.

Depths for rate measurements and kinetic determinations were selected based primarily on PAR and location of the depth of the DCM. High vertical resolution profiles with depth intervals of 5–10 m (from DCM to the depth of 1% of surface PAR) and 10–20 m (from surface to DCM and from 1% of surface PAR to 200 m) were conducted. In total, 14–20 depths were sampled for each station in the SCS.

Samples for chemical, biological, and rate measurements were collected from the same cast. Triplicate 150 mL high-density polyethylene (HDPE) Nalgene bottles were used for nutrient collection; 250 mL polycarbonates (PC) Nalgene bottles and 250 mL HDPE Nalgene bottles were filled for NH_4_^+^ oxidation incubations; 1 L PC Nalgene bottles were used for PON concentration measurements and for NH_4_^+^ uptake and NO_3_^−^ uptake incubations (carried out in duplicate for each treatment in the SCS cruise and in triplicate in the NWP cruise). All bottles and equipment were acid washed and rinsed with seawater three times prior to sample collection.

During the SCS cruise, a set of incubations including kinetics tests were carried out on board to estimate in situ rates for nitrification, NH_4_^+^ uptake, and NO_3_^−^ uptake rates. For the NWP cruise, incubations were carried out for nitrification and NH_4_^+^ uptake rates. Different neutral density screens (Lee Filters) were used to simulate the in situ light intensity. Dark incubation was implemented in parallel to examine light effects and to derive daily rate estimates.

For nitrification rates, 1 mL of ^15^N-NH_4_^+^ tracer (98% of ^15^N atom, Sigma-Aldrich) was injected into 250 mL samples to get a final tracer concentration of 20 nmol L^−1^. Immediately after tracer injection, around 40 mL of sample was filtered through a 0.2 μm syringe filter to represent the initial condition. Paired incubations under simulated in situ light and dark conditions allowed us to properly integrate the daily nitrification rates, and were conducted on-board in on-deck circulating seawater incubators for 12 h (0700–1900 hours) and stopped by filtration. For NH_4_^+^ uptake rates, ^15^N−NH_4_^+^ tracer (98% of ^15^N atom, Sigma-Aldrich) was injected into 1 L samples to get a final concentration of 10 nmol L^−1^. Two to three bottles were filtered (with pressure of ~200 mmHg) onto pre-combusted (450 °C, 4 h) 25 mm glass fiber filters (GF/F, Whatman) immediately after tracer injection to represent the initial conditions. The remaining bottles were incubated for 3 h (0700 to 1000 hours) on board under simulated in situ light and dark conditions in parallel. Similar incubations were conducted for NO_3_^−^ uptake rates with different levels of tracer enrichment. For samples in the mixed layer, the final concentration of ^15^N−NO_3_^−^ (98% of ^15^N atom, Sigma-Aldrich) was 10 nmol L^−1^, and final tracer concentrations were 100 and 500 nmol L^−1^ for samples from the bottom of mixed layer to the DCM and below the DCM, respectively. Incubations were terminated by gentle filtration.

To investigate the dependence of nitrification and NH_4_^+^ uptake rates on substrate concentrations, samples from selected depths were incubated at four different levels of ^15^NH_4_^+^ addition spanning from 10 to 2000 nmol L^−1^ during the SCS cruise. At each station, three depths (15 m, depth of DCM, and depth of 1% PAR) were sampled to conduct Michaelis−Menten kinetic tests. For each set, tracer was added separately into duplicate samples, and all the samples were simultaneously incubated using the same procedures described above.

### Chemical measurements

Oxygen concentrations were measured using an SBE oxygen sensor, calibrated against discrete sample Winkler titrations. NH_4_^+^ concentrations were measured on board using a fluorometric method with detection limit of 1.2 nmol L^−1^ and precision of ±3.5%^57^. Nutrient concentrations below the nitracline were measured using a Four-channel Continuous Flow Technicon AA3 Auto-Analyzer. The detection limits for NOx (NO_3_^−^ + NO_2_^−^), SRP (soluble reactive phosphate), and Si(OH)_4_ were 0.03, 0.03, 0.05 μmol L^−1^, respectively, with precision better than 1, 2, 2.8%, respectively^58^. For surface samples above the nitracline, NO_3_^−^ and NO_2_^−^ concentrations were determined by the standard colorimetric method coupled with a Flow Injection Analysis-Liquid Waveguide Capillary Cell system (World precision Instruments)^59^; the detection limit was 5 nmol L^−1^ and precision was better than 3.1%.

### Isotopic analyses of NO_X_^−^ and PON

δ^15^N of NO_X_^−^ (NO_2_^−^ + NO_3_^−^) were determined using the bacterial method^60, 61^ with minor modifications, using a Thermo Finnigan Gasbench system with cryogenic extraction and purification system interfaced to a Delta V^PLUS^ isotopic ratio mass spectrometer. Briefly, NO_X_^−^ was quantitatively converted to N_2_O by using the bacterial strain *Pseudomonas aureofaciens* (ATTC no. 13985). The N_2_O was then introduced to the GC-IRMS through an online N_2_O cryogenic extraction and purification system. δ^15^N of NO_X_^−^ values were calibrated against nitrate isotope standards USGS 34, IAEA N3 and USGS 32, which were run before, after, and at ten sample intervals. Accuracy (pooled standard deviation) was better than ±0.2‰ according to analyses of these standards at an injection level of 20 nmol N over the past 3 years. Quality control was also conducted by analyzing laboratory working reference material (3000 m deep sea water from the South China Sea). For samples with NO_X_^−^ concentrations lower than 0.5 μmol L^−1^, 1 mL of 5 μmol L^−1^ of in-house NO_3_^−^ standard was added as carrier to 9 mL of samples, and the isotopic composition of the sample was than calculated from the measured composition of the mixture and the known in-house standard via mass conservation.

For the particulate samples, the wet digestion method was applied prior to the bacterial method according to previous studies^62^ with slight modification. In brief, the PON (with filters) was oxidized to NO_3_^−^ by using 1 mL of purified persulfate oxidizing reagent (POR) and 4 mL of deionized water (DIW) in a 12 mL 450 °C pre-combusted boro-silicate glass tube. The persulfate (ACS-grade, Merck, German) was recrystallized at least three times and then made up to alkaline POR by dissolving 6 g K_2_S_2_O_8_ and 6 g NaOH (ACS-grade, Merck, German) in DIW to final volume of 100 mL. Before the use of POR, the residual NO_3_^−^ concentration in the initial POR (referred as POR blank) was measured and ensured to be sufficiently low (<2 μmol L^−1^ in digested solution, see below). Screw caps were closed tightly once the POR was injected into the samples, which were then autoclaved for 1 h under 120 °C. At least five tubes went through the same procedure in parallel with solely POR to determine the δ^15^N of the post-digestion POR blank. In addition, ten unused filters were randomly selected after each cruise to estimate the concentration and δ^15^N of the background PON, i.e. the filter blanks. After the digested solutions cooled down to room temperature, pH was adjusted to neutral with 6 n HCl (ACS-grade, Merck).

The NO_3_^−^ concentration after digestion was measured by chemiluminescene^63^. By injecting 10–1000 μL of sample into a 95 °C acidic solution of vanadium (V(III)), a sufficient amount of NO_3_^−^ was reduced to NO gas for detection. δ^15^N values of the PON-derived NO_3_^−^ were determined using the bacterial method as described above. Calculation of the concentration and δ^15^N of the PON included separate correction for both the blank of the POR procedure and the filters. The PON blank in each tube was typically less than 1% of the total N and the blank of the filter was less than 5 nmol N (1.88 ± 0.85 nmol N after the SCS cruise, and 3.99 ± 1.49 nmol N after the NWP cruise), which accounted for less than 3% of the N content in our samples.

### Rate calibration

Nitrification and uptake rates were primary determined by the accumulation of ^15^N in the product pool relative to the initial conditions. The equations for the rate calculations have been previously reviewed and compared^64^. Equation 1 quantifies the transformation rate under bulk substrate concentration (ambient substrate + tracer). The 10% tracer enrichment principle to minimize tracer perturbation is impractical in oligotrophic oceans with extremely low NH_4_^+^ concentrations^65^. To date, a wide range (10–750 nmol L^−1^) of tracer concentrations has been adopted in different studies^4–10, 24, 25, 30, 66, 67^, and given that NH_4_^+^ concentrations are low across most of global ocean, has likely caused overestimation of measured rates to a certain degree. Similar to other studies, the tracer addition in this study (even though the amount was as low as 10–20 nmol L^−1^) represents a significant substrate enrichment relative to ambient concentrations. Thus, the tracer addition potentially stimulates the in situ rate (Eq. 1).

A few attempts to overcome the enhancement of in situ rate by tracer enrichment have been carried out in the field. By using a kinetic approach (Eq. 2), for instance, Horak et al.^25^ demonstrated severe overestimations of in situ NH_4_^+^ oxidation rates (by factors of 1.1 up to 7.1) with addition of 50 nmol L^−1^ of ^15^NH_4_^+^ into samples at ambient concentrations of 5–650 nmol L^−1^. This conclusion was drawn by assuming a constant *K*_s_ over depth and among sites for NH_4_^+^ oxidation; yet, *K*_s_ may vary with environmental conditions such as temperature and pH. Although the kinetic approach has been suggested as an effective method with better accuracy in calibrating in situ rates, this method has been less commonly conducted due to its labor-consuming requirements.

Instead, because the reaction rate showed a near first-order response when substrate concentration was lower than *K*_s_, we applied a linear regression approach by using Eqs. 3–5 with the following assumptions to obtain the optimal in situ reaction rates: first, the ^15^N dilution effect due to regeneration is insignificant due to relatively short-term incubations, and the isotopic fractionation during N transformation can be ignored because of the high ^15^N enrichment in the substrate after tracer addition, and thus the at% ^15^N of substrate remained constant during the incubation. This is also justified by a recent model study that showed the dilution effect is not significant within 12 h in oligotrophic conditions^68^; second, the at% of ^15^N of in situ NH_4_^+^ was assumed to be 0.3663%.

1 $$R_{{\rm bulk}} = \frac{{C_0 \times (n_t - n_0)}}{{t \times f^{15}}},$$

2 $$R_{{\rm kinetic}} = \frac{{V_{\max} \times Ci_{{\rm NH_4^ +}}}}{{K_{\rm s} + Ci_{{\rm NH_4^ +}}}},$$

3 $$R_{15} = \frac{{C_t \times n_t - C_0 \times n_0}}{t},$$

4 $$R_{14} = R_{15} \times \frac{{(1 - n_{{\rm NH_4^ +}})}}{{n_{{\rm NH_4^ +}}}},$$

5 $$R_{{\rm in}\;{\rm situ}} = (R_{15} + R_{14}) \times \frac{{Ci_{{\rm NH_4^ +}}}}{{Ci_{{\rm NH_4^ +}} + Ct_{{\rm NH_4^ +}}}}.$$

In the above equations, *R*_bulk_ is the bulk reaction rate for all substrates after tracer enrichment (nmol N L^−1^ h^−1^). *R*_kinetic_ is the rate of kinetic response. *R*_15_, *R*_14_ are the accumulation rates of atom ^15^N and ^14^N, respectively, in the product pool. *R*_in situ_ is the in situ reaction rate calibrated by linear interpolation. *C*_*t*_ and *C*_0_ are product concentrations at the ending and beginning of incubation (nmol N L^−1^), respectively. *f*^15^ is at% ^15^N of substrate pool at the beginning of incubation; *n*_*t*_ and *n*_0_ are the at% ^15^N of the product pool at the ending and beginning of incubation (%), respectively. *t* is the duration of incubation (h). $n_{\rm {NH}_{4}^ +}$ is the at% ^15^N of substrate after tracer injection. ${Ci_{\rm {NH}_{4}^+}}$ and ${Ct_{\rm {NH}_{4}^ +}}$ are the initial substrate concentration and final tracer concentration, respectively.

In addition, instead of assuming a constant product concentration during incubation as in most of previous studies, all particle samples at different sampling times were measured directly. This concentration measurement was necessary for uptake rate quantification, because phytoplankton simultaneous uptake of different species of nitrogen may induce considerable increases in PON concentrations during incubation. For NH_4_^+^ oxidation, the calculated rate was converted to the daily-integrated rate by assuming equal periods of day and night rates, i.e. by summing the dark and light hourly rates multiplied by 12 (*R*_in situ of light incubation_ × 12 + *R*_in situ of dark incubation_ × 12); daily-integrated NH_4_^+^ and NO_3_^−^ uptake rate was approximately estimated by multiplying the hourly rates by 16 according to previous tests at the Bermuda Atlantic Time Series station (BATS)^5^.

In order to test the validity of the linear regression approach presented here, we compared the *R*_bulk_, *R*_in situ_ and *R*_kinetic_ derived from the above equations (Supplementary Fig. 10) by using the data obtained from kinetic experiments. The NH_4_^+^ concentrations of 21–94 nmol L^−1^ (in situ NH_4_^+^ + tracer) were lower than the derived *K*_s_ values in this study, allowing the application of the proposed linear regression method for in situ rate calibration. Our results showed much higher values of *R*_bulk_ relative to *R*_kinetic_, demonstrating significant overestimation of reaction rates by tracer addition. The reaction rates were reduced by 15.2–78.4% by using the kinetic correction. Larger deviations were found at depths with low in situ concentrations. By contrast, the *R*_in situ_ derived from the linear regression was close to that of *R*_kinetic_, demonstrating the usefulness of linear interpolation for in situ rates determination. Further, given the persistent low NH_4_^+^ concentration in the global oligotrophic ocean, this method offers a convenient approach to correct reaction rates derived from tracer incubations.

### Detection limit of rate measurement

The detection limit depends on the concentration of the product pool and the fraction of the ^15^N in the substrate pool during the incubation^24, 66, 67^. As mentioned, the accuracy of δ^15^-NO_3_^−^ was better than ±0.2‰ in our lab, and we here use three times the standard deviation (0.6‰) as a reliable enrichment of ^15^N in the product pool. Therefore, we calculated a detection limit of 0.04–0.16 nmol N L^−1^ d^−1^ for nitrification and 0.01–0.04 nmol N L^−1^ d^−1^ for NH_4_^+^ uptake for our samples.

### Data analysis

The kinetic parameters (*V*_max_ and *K*_s_) were estimated using Eq. 2 (and Grapher version 10; Golden Software). The *α* value, which represents a better indicator of advantage in substrate competition^12, 17^, was calculated by using Eq. 6.

6 $${{\alpha }} = \frac{{V_{\max}}}{{K_{\mathrm s}}}.$$

The comparisons of reaction rates between paired light and dark incubations, and the kinetic parameters were examined by using the Student’s *t *test and SPSS (IBM, version 19).

### Data availability

Data supporting our manuscript are available on request from S.-J. Kao (sjkao@xmu.edu.cn).

## Electronic supplementary material

Supplementary Information

## References

[1] Eppley RW, Peterson BJ (1979). Particulate organic matter flux and planktonic new production in the deep ocean. Nature.

[2] Olson RJ (1981). Differential photoinhibition of marine nitrifying bacteria: a possible mechanism for the formation of the primary nitrite maximum. J. Mar. Res..

[3] Horrigan SG, Carlucci AF, Williams PM (1981). Light inhibition of nitrification in sea-surface films [California]. J. Mar. Res..

[4] Beman JM (2011). Global declines in oceanic nitrification rates as a consequence of ocean acidification. Proc. Natl. Acad. Sci. USA.

[5] Lipschultz F (2001). A time-series assessment of the nitrogen cycle at BATS. Deep-Sea Res. II.

[6] Shiozaki T (2016). Nitrification and its influence on biogeochemical cycles from the equatorial Pacific to the Arctic Ocean. ISME J..

[7] Ward BB (2005). Temporal variability in nitrification rates and related biogeochemical factors in Monterey Bay, California, USA. Mar. Ecol. Prog. Ser..

[8] Smith JM, Chavez FP, Francis CA (2014). Ammonium uptake by phytoplankton regulates nitrification in the sunlit ocean. PLoS ONE.

[9] Lomas MW (2009). Biogeochemical responses to late-winter storms in the Sargasso Sea, I—pulses of primary and new production. Deep-Sea Res. I.

[10] Newell SE, Fawcett SE, Ward BB (2013). Depth distribution of ammonia oxidation rates and ammonia-oxidizer community composition in the Sargasso Sea. Limnol. Oceanogr..

[11] Finkel ZV (2010). Phytoplankton in a changing world: cell size and elemental stoichiometry. J. Plankton Res..

[12] Lomas MW, Bonachela JA, Levin SA, Martiny AC (2014). Impact of ocean phytoplankton diversity on phosphate uptake. Proc. Natl. Acad. Sci. USA.

[13] Irwin AJ, Finkel ZV, Schofield OME, Falkowshi PG (2006). Scaling-up from nutrient physiology to the size-structure of phytoplankton communities. J. Plankton Res..

[14] Marañón E (2015). Cell size as a key determinant of phytoplankton metabolism and community structure. Annu. Rev. Mar. Sci..

[15] Van Oostende N (2017). Variation of summer phytoplankton community composition and its relationship to nitrate and regenerated nitrogen assimilation across the North Atlantic Ocean. Deep-Sea Res. I.

[16] Harrison WG, Harris LR, Irwin BD (1996). The kinetics of nitrogen utilization in the oceanic mixed layer: nitrate and ammonium interactions at nanomolar concentrations. Limnol. Oceanogr..

[17] Healey FP (1980). Slope of the Monod equation as an indicator of advantage in nutrient competition. Microb. Ecol..

[18] Kara AB, Rochford PA, Hurlburt HE (2000). An optimal definition for ocean mixed layer depth. J. Geophys. Res. Oceans.

[19] Aksnes DL, Ohman MD, Rivière P (2007). Optical effect on the nitracline in a coastal upwelling area. Limnol. Oceanogr..

[20] Smith JM, Damashek J, Chavez FP, Francis CA (2016). Factors influencing nitrification rates and the abundance and transcriptional activity of ammonia-oxidizing microorganisms in the dark northeast Pacific Ocean. Limnol. Oceanogr..

[21] Buesseler KO (2008). VERTIGO (VERtical Transport In the Global Ocean): a study of particle sources and flux attenuation in the North Pacific. Deep-Sea Res. II.

[22] Ward, B. B. In *Nitrogen in the Marine Environment* 2nd edn (eds Capone, D. G. et al.) 199–261 (Academic, Burlington, MA).

[23] Buchwald C, Casciotti KL (2013). Isotopic ratios of nitrite as tracers of the sources and age of oceanic nitrite. Nat. Geosci..

[24] Santoro AE (2013). Measurements of nitrite production in and around the primary nitrite maximum in the central California Current. Biogeosciences.

[25] Horak REA (2013). Ammonia oxidation kinetics and temperature sensitivity of a natural marine community dominated by Archaea. ISME J..

[26] Levipan HA (2016). Variability of nitrifying communities in surface coastal waters of the Eastern South Pacific (approximately 36 degrees S). Env. Microbiol. Rep..

[27] Kim J (2016). Hydrogen peroxide detoxification is a key mechanism for growth of ammonia-oxidizing archaea. Proc. Natl. Acad. Sci. USA.

[28] Luo H (2014). Single-cell genomics shedding light on marine Thaumarchaeota diversification. ISME J..

[29] Santoro AE (2015). Genomic and proteomic characterization of “Candidatus Nitrosopelagicus brevis”: an ammonia-oxidizing archaeon from the open ocean. Proc. Natl. Acad. Sci. USA.

[30] Beman JM, Popp BN, Francis CA (2008). Molecular and biogeochemical evidence for ammonia oxidation by marine Crenarchaeota in the Gulf of California. ISME J..

[31] Francis CA, Roberts KJ, Beman JM, Santoro AE, Oakley BB (2005). Ubiquity and diversity of ammonia-oxidizing archaea in water columns and sediments of the ocean. Proc. Natl. Acad. Sci. USA.

[32] Martens-Habbena W, Berube PM, Urakawa H, de al Torre JR, Stahl DA (2009). Ammonia oxidation kinetics determine niche separation of nitrifying Archaea and Bacteria. Nature.

[33] Qin W (2014). Marine ammonia-oxidizing archaeal isolates display obligate mixotrophy and wide ecotypic variation. Proc. Natl. Acad. Sci. USA.

[34] Dortch Q, Thompson PA, Harrison PJ (1991). Short-term interaction between nitrate and ammonium uptake in Thalassiosira pseudonana: effect of preconditioning nitrogen source and growth rate. Mar. Biol..

[35] Yin K, Harrison PL, Dortch Q (1998). Lack of ammonium inhibition of nitrate uptake for a diatom grown under low light conditions. J. Exp. Mar. Biol. Ecol..

[36] Edwards KF, Thomas MK, Klausmeier CA, Litchman E (2015). Light and growth in marine phytoplankton: allometric, taxonomic, and environmental variation. Limnol. Oceanogr..

[37] Thompson PA, Levasseur ME, Harrison PJ (1989). Light-limited growth on ammonium vs. nitrate: what is the advantage for marine phytoplankton?. Limnol. Oceanogr..

[38] Painter SC, Patey MD, Tarran GA, Torres-Valdés S (2014). Picoeukaryote distribution in relation to nitrate uptake in the oceanic nitracline. Aquat. Microb. Ecol..

[39] Fawcett SE, Lomas MW, Casey JR, Ward BB, Sigman DM (2011). Assimilation of upwelled nitrate by small eukaryotes in the Sargasso Sea. Nat. Geosci..

[40] Li WKW (2002). Macroecological patterns of phytoplankton in the northwestern North Atlantic Ocean. Nature.

[41] Chen B (2011). Comparisons of picophytoplankton abundance, size, and fluorescence between summer and winter in northern South China Sea. Cont. Shelf Res..

[42] Liu H, Chang J, Tseng CM, Wen LS, Liu KK (2007). Seasonal variability of picoplankton in the Northern South China Sea at the SEATS station. Deep-Sea Res. II.

[43] Campbell L, Liu H, Nolla HA, Vaulot D (1997). Annual variability of phytoplankton and bacteria in the subtropical North Pacific Ocean at Station ALOHA during the 1991–1994 ENSO event. Deep-Sea Res. I.

[44] DuRand MD, Olson RJ, Chisholm SW (2001). Phytoplankton population dynamics at the Bermuda Atlantic Time-series station in the Sargasso Sea. Deep-Sea Res. II.

[45] Casey JR, Lomas MW, Mandechi J, Walker DE (2007). Prochlorococcus contributes to new production in the Sargasso Sea deep chlorophyll maximum. Geophys. Res. Lett..

[46] Martiny AC, Kathuria S, Berube PM (2009). Widespread metabolic potential for nitrite and nitrate assimilation among Prochlorococcus ecotypes. Proc. Natl. Acad. Sci. USA.

[47] Edwards KF, Thomas MK, Klausmeier CA, Litchman E (2012). Allometric scaling and taxonomic variation in nutrient utilization traits and maximum growth rate of phytoplankton. Limnol. Oceanogr..

[48] Litchman E, Klausmeier CA, Schofield OM, Falkowski PG (2007). The role of functional traits and trade-offs in structuring phytoplankton communities: scaling from cellular to ecosystem level. Ecol. Lett..

[49] Carter CM, Ross AH, Schiel DR, Howard-Willams C, Hayden B (2005). *In situ* microcosm experiments on the influence of nitrate and light on phytoplankton community composition. J. Exp. Mar. Biol. Ecol..

[50] Donald DB, Bogard MJ, Finlay K, Bunting L, Leavitt PR (2013). Phytoplankton-specific response to enrichment of phosphorus-rich surface waters with ammonium, nitrate, and urea. PLoS ONE.

[51] Örnólfsdóttir EB, Lumsden SE, Pinckney JL (2004). Nutrient pulsing as a regulator of phytoplankton abundance and community composition in Galveston Bay, Texas. J. Exp. Mar. Biol. Ecol..

[52] Bianchi M, Feliatra, Lefevre D (1999). Regulation of nitrification in the land-ocean contact area of the Rhône River plume (NW Mediterranean). Aquat. Microb. Ecol..

[53] Hsiao SSY (2014). Nitrification and its oxygen consumption along the turbid Chang Jiang River plume. Biogeosciences.

[54] Yool A, Martin AP, Fernandez C, Clark DR (2007). The significance of nitrification for oceanic new production. Nature.

[55] Boyce DG, Lewis MR, Worm B (2010). Global phytoplankton decline over the past century. Nature.

[56] Flombaum P (2013). Present and future global distributions of the marine Cyanobacteria Prochlorococcus and Synechococcus. Proc. Natl. Acad. Sci. USA.

[57] Zhu Y, Yuan D, Huang Y, Ma J, Feng S (2013). A sensitive flow-batch system for on board determination of ultra-trace ammonium in seawater: method development and shipboard application. Anal. Chim. Acta.

[58] Han A (2012). Nutrient dynamics and biological consumption in a large continental shelf system under the influence of both a river plume and coastal upwelling. Limnol. Oceanogr..

[59] Zhang J (2000). Shipboard automated determination of trace concentrations of nitrite and nitrate in oligotrophic water by gas-segmented continuous flow analysis with a liquid waveguide capillary flow cell. Deep-Sea Res. I.

[60] Casciotti KL, Sigman DM, Hastings MG, Böhlke JK, Hilkert A (2002). Measurement of the oxygen isotopic composition of nitrate in seawater and freshwater using the denitrifier method. Anal. Chem..

[61] Sigman DM (2001). A bacterial method for the nitrogen isotopic analysis of nitrate in seawater and freshwater. Anal. Chem..

[62] Knapp AN, Sigman DM, Lipschultz FN (2005). Isotopic composition of dissolved organic nitrogen and nitrate at the Bermuda Atlantic Time-series Study site. Glob. Biogeochem. Cy..

[63] Braman RS, Hendrix SA (1989). Nanogram nitrite and nitrate determination in environmental and biological materials by vanadium (III) reduction with chemiluminescence detection. Anal. Chem..

[64] Ward BB, Kilpatrick KAA, Renger EH, Eppley RW (1989). Biological nitrogen cycling in the nitracline. Limnol. Oceanogr..

[65] Ward, B. B. In *Methods in Enzymology* Vol. 486 (ed. Koltz, M. G.) 307–323 (Academic Press, Burlington, 2011).

[66] Peng X (2015). Ammonia and nitrite oxidation in the Eastern Tropical North Pacific. Glob. Biogeochem. Cy.

[67] Peng X (2016). Revisiting nitrification in the Eastern Tropical South Pacific: a focus on controls. J. Geophys. Res. Oceans.

[68] Xu NM (2017). Quantification of multiple simultaneously occurring nitrogen flows in the euphotic ocean. Biogeosciences.

[69] Bianchi M, Feliatra F, Tréguer P, Vincendeau MA, Morvan J (1997). Nitrification rates, ammonium and nitrate distribution in upper layers of the water column and in sediments of the Indian sector of the Southern Ocean. Deep-Sea Res. II.

