# Supplementary material for: Ambient nitrate switches the ammonium consumption pathway in the euphotic ocean
Source: Nat Commun. 2018 Mar 2;9:915. doi: 10.1038/s41467-018-03363-0 (PMC5834513; doi:10.1038/s41467-018-03363-0)
Supplement: Supplementary file 1 — Supplementary Information [file 41467_2018_3363_MOESM1_ESM.pdf]

Supplementary Information

Ambient nitrate switches the ammonium consumption pathway in the euphotic ocean

Xianhui Sean Wan[1], Hua-Xia Sheng[1], Minhan Dai[1], Yao Zhang[1], Dalin Shi[1], Thomas W. Trull[2], Yifan Zhu[1], Michael W. Lomas[3], Shuh-Ji Kao[1, *]

[1]State Key Laboratory of Marine Environmental Sciences, Xiamen University, Xiamen 361101, China

[2]Antarctic Climate and Ecosystems Cooperative Research Centre, University of Tasmania, and CSIRO Oceans and Atmosphere, Hobart, Australia

[3]Bigelow Laboratory for Ocean Sciences, East Boothbay, ME 04544, USA

**Correspondence to:* sjkao@xmu.edu.cn

Supplementary figures

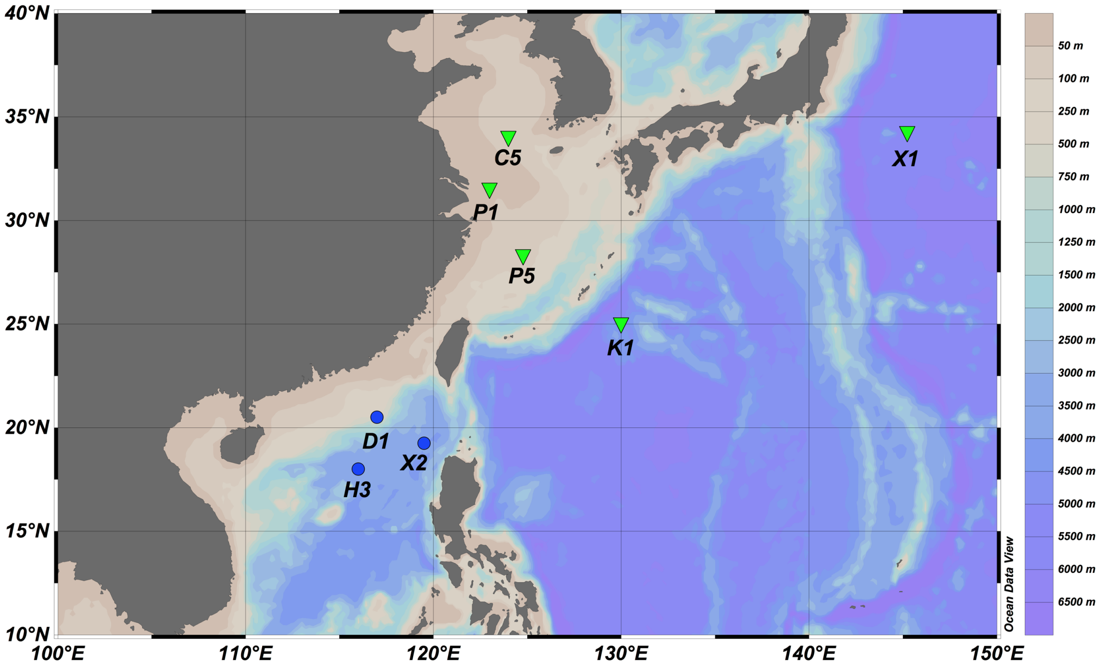

Supplementary Fig. 1 Study area and sampling sites. The blue dots show sampling sites during the South China Sea cruise in 2014; the green triangles show sampling sites in the subtropical Northwestern Pacific in 2015. The figure is created by using Ocean Data View 4.6.3 (http://odv.awi.de).

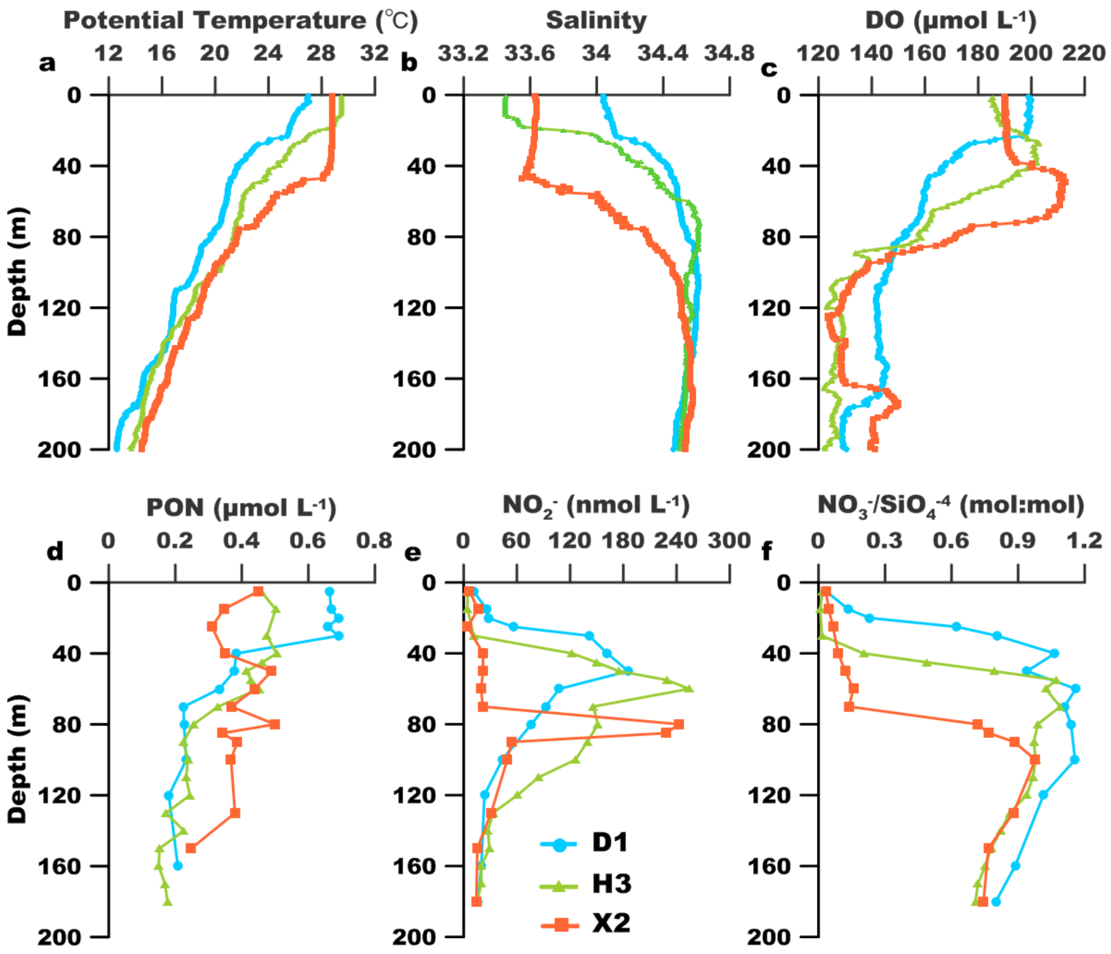

Supplementary Fig. 2 Hydrological and biological characteristics of sampling sites in the South China Sea during 2014. D1: Blue line; H3: Green line and X2: Orange line. a: Potential temperature (C°); b: Salinity; c: Dissolved oxygen (DO, μmol L^{-1}); d: Particulate organic nitrogen (PON, μmol L^{-1}); e: NO_2^- (nmol L^{-1}); f: NO_3^-/SiO_4^{-4} (molar ratio).

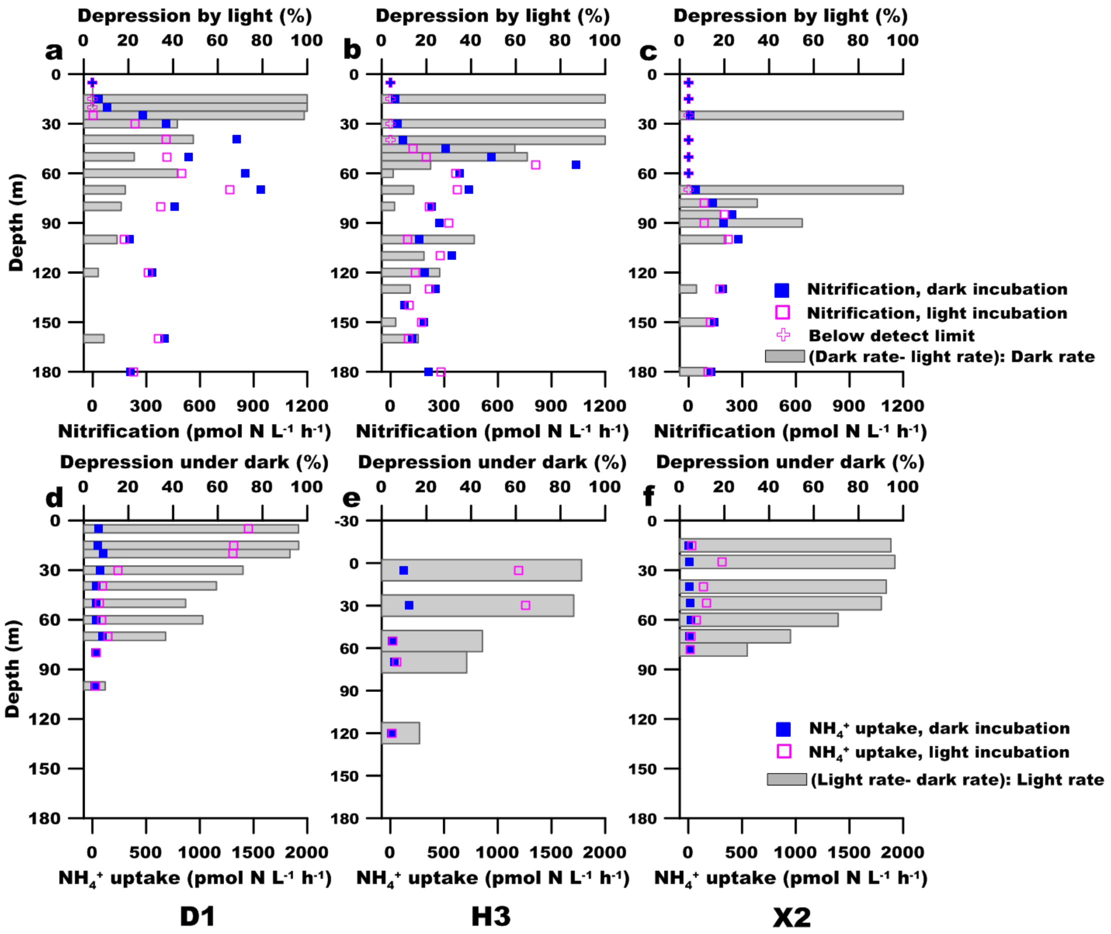

Supplementary Fig. 3 NH_4^+ uptake rates and nitrification rates under light and dark conditions in the SCS. Panels a-c show the nitrification rates and panes d-f show the NH_4^+ uptake rates. Blue solid squares represent average reaction rates of duplicates in the dark; pink open squares represent rates under simulated near *in-situ* light; crosses indicate rates below the detection limits; gray bars in the upper panel show percent depression of nitrification rates under light (that is: (dark rates – light rates)/ dark rates x 100), and the lower panel shows depression of NH_4^+ uptake rates under dark (that is: (light rates – dark rates)/ light rates x 100). a and d show results at D1, b and e show results at H3, c and f show results at X2.

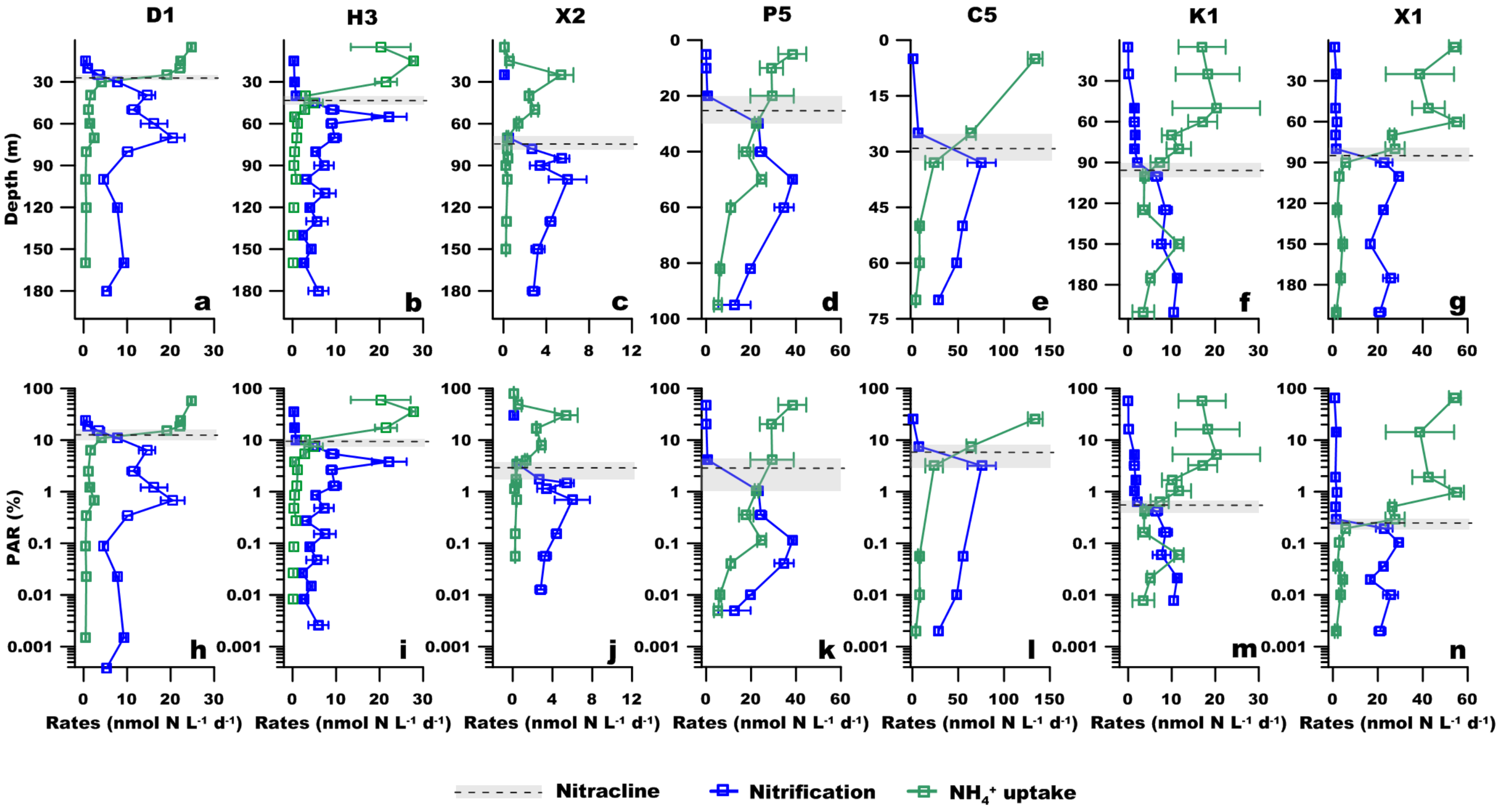

Supplementary Fig. 4 Vertical distribution of nitrification and NH_4^+ uptake rates in the SCS and the subtropical Northwest Pacific. a and h: Station D1; b and i: Station H3; c and j: Station X2; d and k: Station P5; e and l: Station C5; f and m: Station K1; g and n: Station X1. Rates are plotted against depth in the upper panel and against PAR in the lower panel. Gray bars mark the depth range with the steepest NO_3^- gradient and the black dashed lines inside show the nitricline depth (see methods). Error bars of NH_4^+ uptake rate and nitrification rates are represented by the standard deviation of duplicates (for D1, H3 and X2) or triplicates (P5, C5, K1and X1), and are smaller than the symbol where not visible.

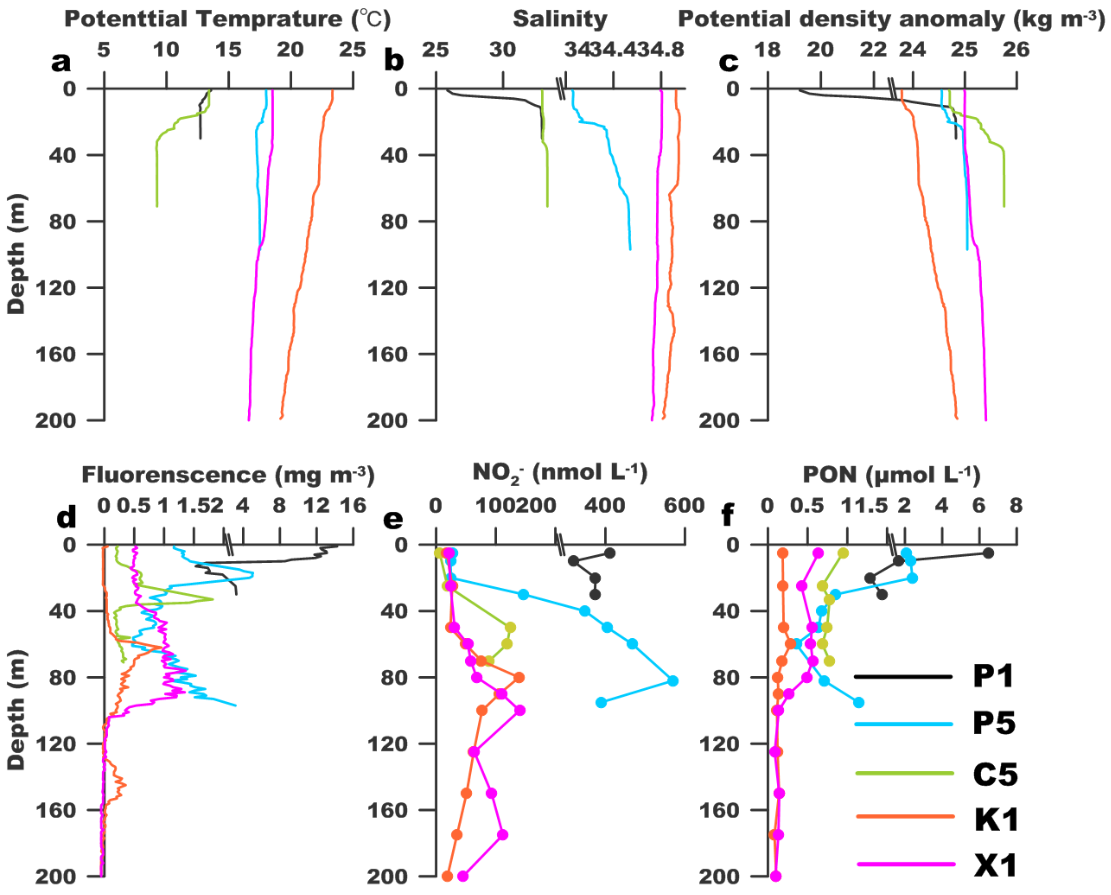

Supplementary Fig. 5 Hydrological and biological characteristics of sampling sites in the subtropical Northwest North Pacific during 2015. P1: Black line; P5: Blue line; C5: Green line; K1: Orange line and X1: Pink line. a: Potential temperature (C°); b: Salinity; c: Potential density anomaly (kg m^{-3}); d: Fluorescence (mg m^{-3}); e: NO_2^- (nmol L^{-1}); f: PON (μmol L^{-1}).

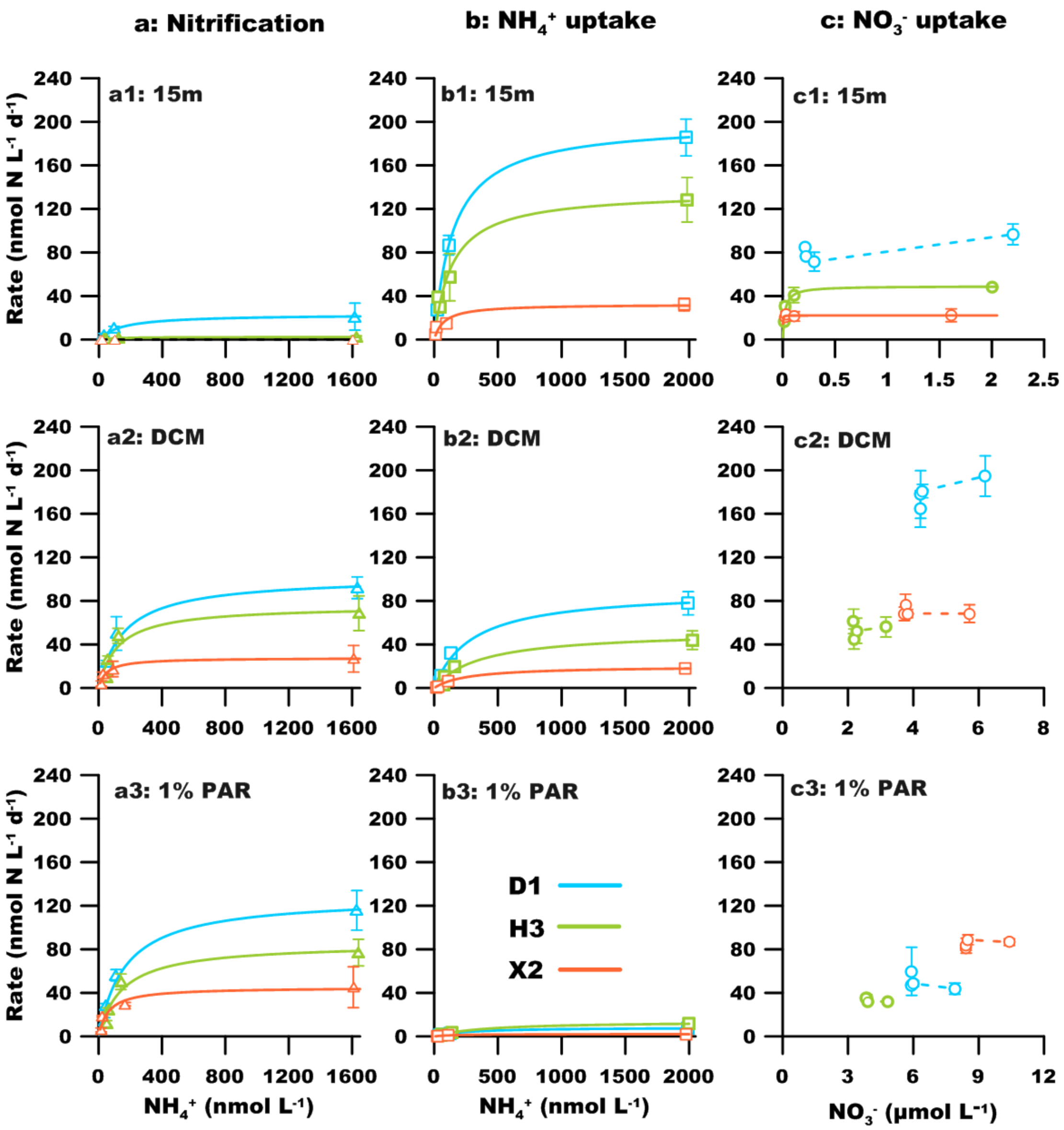

Supplementary Fig. 6 Reaction rates of nitrification, NH_4^+ uptake and NO_3^- uptake under different substrate enrichment in the SCS. The solid lines were fit using the Michaelis-Menten equation; dashed lines indicate reactions without kinetic responses. Triangles are nitrification rates (Column a), squares are NH_4^+ uptake rates, and circles are NO_3^- uptake rates (Column b and c). The blue, green and orange colors indicate stations D1, H3 and X2, respectively. Reaction rates are shown at 15m (upper panels); in the DCM layer (middle panels), and at the depth of 1% PAR (lower panels). Error bars represent the standard deviation of two replicates (±1σ), and are smaller than the symbols where not visible.

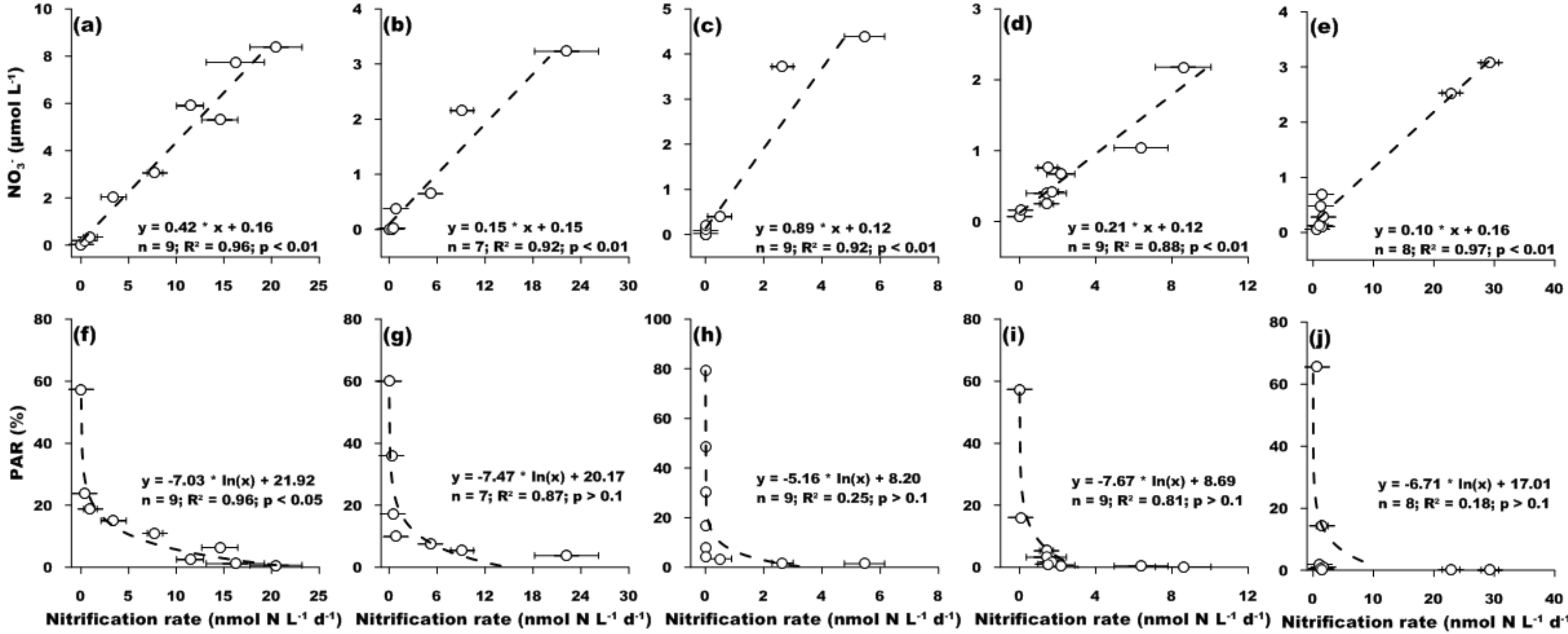

Supplementary Fig. 7 Correlation between nitrification rates, NO_3^- concentrations and light levels from surface to the depth of rate maxima. The relationship between nitrification and NO_3^- concentration was best fit by using linear regression (a - e), and the relationship between nitrification and PAR by logarithmic regression (f - j). Panels a and f, b and g, c and h, d and i, e and j show the results of D1, H3, X2, K1, X1 station, respectively. Error bars represent the standard deviation of two replicates (±1σ), and are smaller than the symbols where not visible.

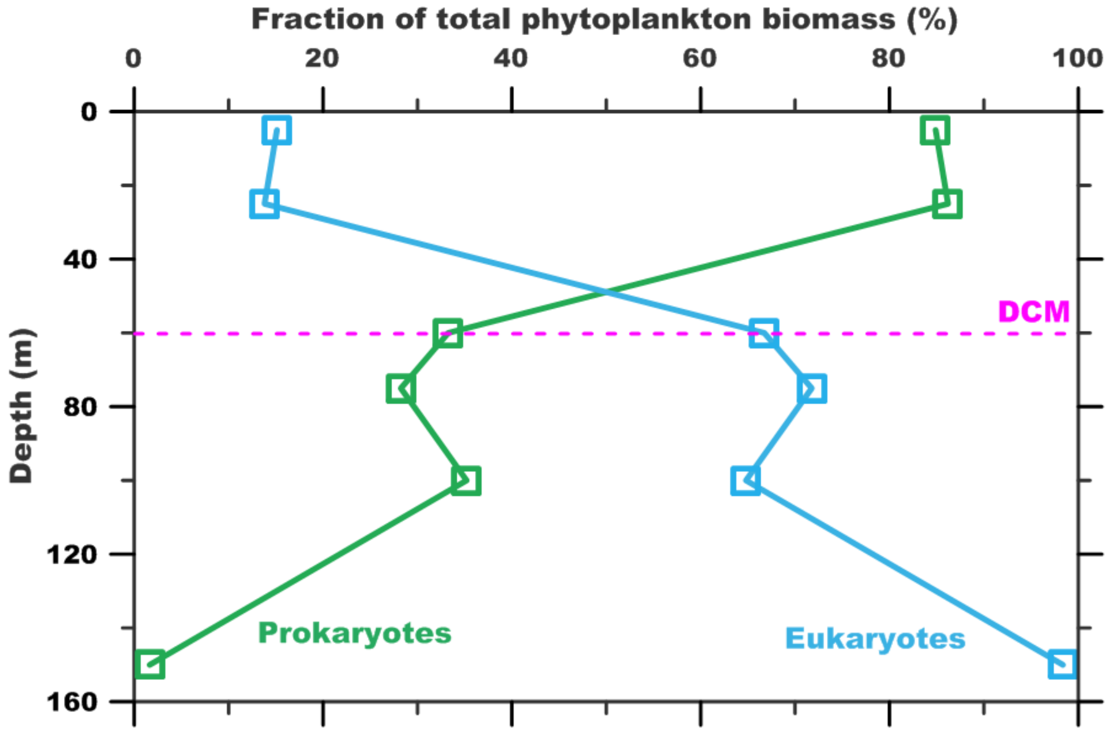

Supplementary Fig. 8 The contributions of prokaryotic and eukaryotic biomass to total phytoplankton biomass in the euphotic zone of H3 station. The blue squares represent eukaryotes and the green squares represent prokaryotes. Biomasses were derived from HPLC pigments data by using the chemical taxonomy program, CHEMTAX. The pink dashed line marks the location of the DCM (Data from: Huang et al. unpublished data, with permission).

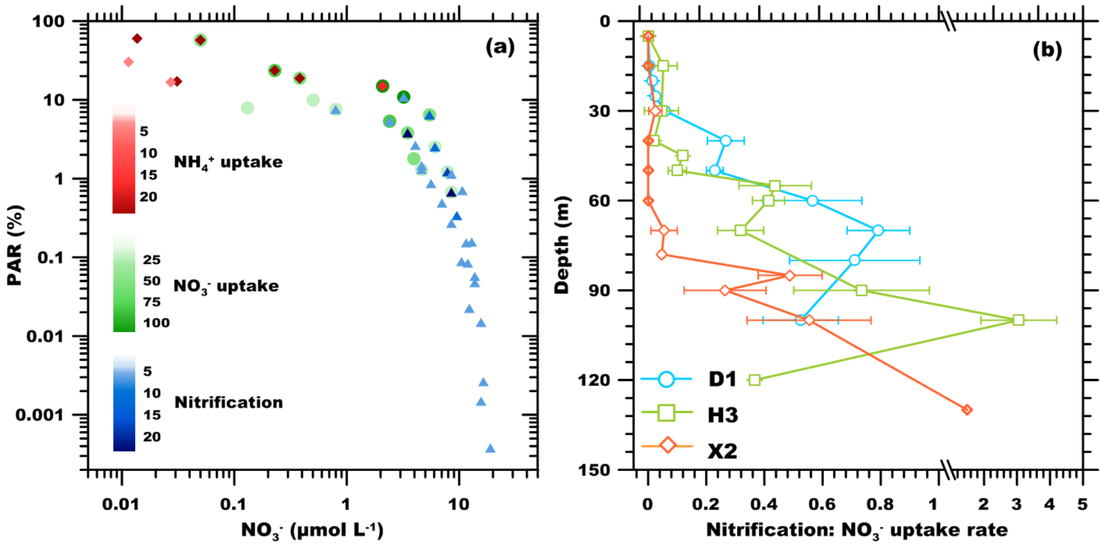

Supplementary Fig. 9 Distributions of NH_4^+ uptake, NO_3^- uptake and nitrification relative to nitrate and light levels, and the contribution of regenerated NO_3^- to new production in the SCS. a: NH_4^+ uptake rates (diamond), NO_3^- uptake rates (circle) and nitrification rates (triangle) against PAR and NO_3^-. Color bars show rates of each process (nmol N L^{-1} d^{-1}). b: ratio of nitrification rates to NO_3^- uptake rates in the euphotic zone. Stations D1, H3, and X2 are shown in blue, green and orange, respectively.

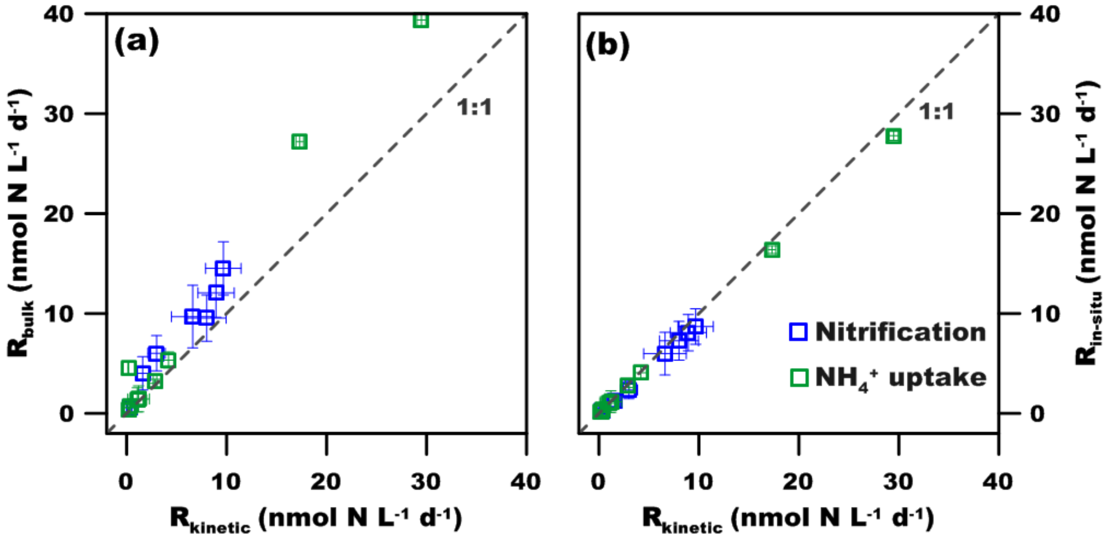

Supplementary Fig. 10 Comparison of NH_4^+ uptake rates and NH_4^+ oxidation rates derived from different approaches. a: Bulk rates (R_{bulk}, derived from Equation S1) versus the rates derived from kinetic determination ($R_{kinetic}$, Equation S2); b: rates derived from our regression approach ($R_{in\text{-}situ}$, Equation S5) versus $R_{kinetic}$ values. Blue squares are nitrification rates and green squares are NH_4^+ uptake rates. The dashed gray line has a slope of 1. Error bars represent the standard deviation of two replicates (±1σ), and are smaller than the symbols where not visible.

Supplementary tables

Supplementary Table 1. Significance of reaction rate differences for the light and dark incubations in the SCS. Rates were compared using the student's *t*-test.

Sites	Nitrification	NH_4^+ uptake
D1	n = 12, p = 0.004	n = 9, p = 0.053
H3	n = 15, p = 0.024	n = 5, p = 0.169
X2	n = 9, p = 0.008	n = 7, p = 0.050

Supplementary Table 2. Significance of kinetic parameter differences between NH_4^+ uptake and nitrification. Values were compared using the student's *t*-test.

Sites	V_{max}	K_s	α
Above the nitracline	n = 3, p = 0.116	n = 3, p = 0.191	n = 3, p = 0.063
Below the nitracline	n = 6, p = 0.046	n = 6, p = 0.002	n =6, p =0.001

Supplementary Table 3. Significance of correlations between NO_3^- uptake rates and chlorophyll fluorescence in the SCS.

Sites	n	R^2	p
D1	13	0.80	0.001
H3	15	0.67	0.005
X2	13	0.54	0.053
All	41	0.63	0.000

Supplementary Note 1: Inactive nitrification above the nitracline

In many cases, the mixed layer and nitricline are deeper than the euphotic zone, because the density difference at the base of the mixed layer impedes supply of subsurface NO_3^-. Lomas et al. (2009) and Newell et al. (2013) found that nitrification in the entire euphotic zone was inactive in the Sargasso Sea when the deep nitracline formed during wintertime[1, 2]. The whole phytoplankton community living above the deep nitracline showed complete reliance on recycled N during their observing period[3]. Consequently, more intensive competition for NH_4^+ by phytoplankton leaves less substrate for NH_4^+ oxidizers in the water column despite the reduction of light inhibition of NH_4^+ oxidation during wintertime due to lower seasonal irradiance.

In summertime, when the mixed layer shoals to the upper euphotic zone, much higher euphotic zone nitrification rates have been detected. Concomitant elevation in the $\delta^{15}N$ of small eukaryotic phytoplankton[4] suggests increased consumption of diapycnal supplied NO_3^- and thus that this shift in nitrogen reliance alleviates substrate limitation for NH_4^+ oxidizers. Therefore, the seasonal variation in nitrification intensity is largely influenced by physical processes that regulate nutrient sources for phytoplankton, the vertical distribution of phytoplankton community, and thus the competition with nitrifier for ammonium.

Supplementary references

1. Lomas, M. W., Lipschultz, F., Nelson, D. M., Krause, J. W. & Bates, N. R. Biogeochemical responses to late-winter storms in the Sargasso Sea, I—Pulses of primary and new production. *Deep-Sea Res. I* **56,** 843-860 (2009).
2. Newell, S. E., Fawcett, S. E. & Ward, B. B. Depth distribution of ammonia oxidation rates and ammonia-oxidizer community composition in the Sargasso Sea. *Limnol. Oceanogr.* **58,** 1491-1500 (2013).
3. Fawcett, S. E., Lomas, M. W., Ward, B. B. & Sigman, D. M. The counterintuitive effect of summer-to-fall mixed layer deepening on eukaryotic new production in the Sargasso Sea. *Global Biogeochem. Cy.* **28,** doi:10.1002/ 2013GB004579 (2014).
4. Treibergs, L. A., Fawcett, S. E., Lomas, M. W. and Sigman, D. M. Nitrogen isotopic response of prokaryotic and eukaryotic phytoplankton to nitrate availability in Sargasso Sea surface waters. *Limnol. Oceanogr.* **59,** 972-985 (2014).
